# Fungal Infection among Diabetic and Nondiabetic Individuals in Nepal

**DOI:** 10.1155/2020/7949868

**Published:** 2020-11-18

**Authors:** Bhuvan Saud, Prajuna Bajgain, Govinda Paudel, Vikram Shrestha, Dipendra Bajracharya, Saroj Adhikari, Gunaraj Dhungana, Mamata Sherpa Awasthi

**Affiliations:** ^1^Department of Medical Laboratory Technology, Janamaitri Foundation Institute of Health Sciences, Hattiban, Lalitpur, Nepal; ^2^Tribhuvan University Teaching Hospital, Institute of Medicine, Kathmandu, Nepal; ^3^Pyuthan District Hospital, Pyuthan, Nepal; ^4^Department of Nursing, Janamaitri Foundation Institute of Health Sciences, Hattiban, Lalitpur, Nepal

## Abstract

**Background:**

Low immunity, comorbid clinical conditions, and metabolic disorders may be the underlying factors that determine the severity of infection. Diabetes increases the risk of infection and multiple organ damage. In Nepal, the actual burden of fungal infections has not been estimated or is in a limited progress. This study aimed to investigate the status of fungal infection in diabetic and nondiabetic individuals in Bhaktapur, Nepal.

**Materials and Methods:**

A total of 670 samples were collected from 134 participants. From each participant, five samples were collected from different sites like an oral wash, toe swab, midstream urine, hair shaft, and nail scrapings. All samples were cultured on Sabouraud dextrose agar. Gram stain was used to observe yeast cells and lactophenol cotton blue stain was used for hyphae. Chlamydospore production by *Candida* species was observed in cornmeal agar medium by Dalmau Plate method. *Candida* species isolated were characterized by germ-tube test and differentiated using CHROM agar *Candida* medium. *Candida* species isolates were tested for antibiotic susceptibility.

**Results:**

Overall, 19.4% of the samples showed fungal growth. The prevalence of fungal infection was higher in diabetic (34.0%) than nondiabetic individuals (4.7%). Fungal growth was found to be higher in oral wash followed by toe, urine, hair, and nail samples. Predominant fungi were *Candida* species (57.5%), *Aspergillus* species (28.4%), and *Trichophyton* species (10.7%). Oral wash, toe, and urine samples in diabetics had a significantly higher fungal prevalence when compared between both groups, *p* value < 0.05. In *Candida* isolates, higher resistance was seen against fluconazole 36.8% and ketoconazole 28.9%, whereas other drugs showed low resistance.

**Conclusion:**

Diabetic participants are more susceptible to fungal infection than the nondiabetics. Overall, *Candida* species and *Aspergillus* species are highly predominant fungi. *Candida* species are highly resistant to fluconazole and ketoconazole.

## 1. Introduction

Fungal infections are taken less seriously but these are present as silent killers. Globally, more than 300 million people are at extremely high risk and 25 million people are at high risk of dying due to fungal infections [1]. Diabetes is an endocrine disorder. Patients of uncontrolled diabetes are susceptible to infection due to metabolic disorder, xerostomia, immune-related dysfunctions, and multiple organ disorders. Annually, around 422 million people are affected by diabetes, and the burden is high in low and middle income countries [2]. In Nepal, due to diabetes, the proportional mortality rate has been found to be 3.0% in all ages and its prevalence is much higher in male than female, that is, 10.5% and 7.9%, respectively, as of 2016 [3], which increased up to 3.97% in 2017 [4]. Diabetes leads to the development of opportunistic infections in the skin, foot, urinary tract, surgical sites, and so on through affecting the immune system. In addition, fungal infections have become more severe in the patients with HIV/AIDS, malignancy, and transplant and those who are under steroids therapy and with other immunosuppressive conditions as well [5, 6].

The clinical severity of fungal infections ranges from asymptomatic to mild skin infections to serious invasive infections. According to the Global Action Fund for Fungal Infectious (GAFFI), annually, about 135 million women are affected by vulvovaginal candidiasis (thrush), almost 1 million people are affected by invasive candidiasis, 60,000–100,000 cases of *Candida* peritonitis occur, above 300,000 patients develop invasive aspergillosis, 400,000 cases of pneumocystis pneumonia are seen, and about 500,000 new infections of histoplasmosis exist globally [7]. Cutaneous fungal infections in the skin, hair, and nails affect around 1 billion people [8]. It was reported that 1.87% of Nepalese population suffer from serious fungal infection and most infections are keratitis (73 per 100,000 annually), invasive mycoses (1119 cases annually), and bronchopulmonary aspergillosis (2673–13 364), pneumocystis pneumonia (990 cases annually), oral infection (10,347 cases), and oesophageal candidiasis (2,950 cases) [5]. Candidiasis is a very common mycotic infection around the world and *Candida* albicans is the most common etiological agent of candidiasis. Other species such as *C. glabrata*, *C. krusei*, and *C. tropicalis* are opportunistic pathogens [9]. Epidemiological study showed that *Aspergillus* species including *A. flavus*, *A. niger*, and *A. fumigatus* cause infection in nails, ears, eyes, respiratory tract, and skin [5]. The morbidity and mortality of infection is much higher in patients with other comorbid clinical conditions, like immunological impairment, chemotherapy, cancer, and long-term chronic diseases.

Uncontrolled hyperglycemic condition leads to infection due to dysfunction of the immune system by the reduction of T-lymphocyte, neutrophil activity, reduction of secretion of inflammatory cytokines, disorders of antibody mediated immunity along with angiopathy, neuropathy, glycosuria, and increased apoptosis of polymorphonuclear leukocytes [10]. It has been observed that diabetic patients show polymicrobial growth of pathogens. Several studies have shown that the prevalence of fungi ranges from 7.0 to 17.38% in diabetic patients, and fungal species including *Candida* species, *Aspergillus* species, *Fusarium* species, *Rhodotorula* species, and *Trichosporon* species are the most frequent [11–13]. However, all fungi are not harmful and most of their infections can be managed if diagnosed timely. In our context, mycological studies are limited because of the lack of sophisticated healthcare facilities and awareness in community. Thus, present study is designed to investigate the prevalence of fungi in diabetic and nondiabetic individuals of Bhaktapur district and also to study the antibiotic pattern of isolated *Candida* species.

## 2. Materials and Methods

### 2.1. Study Site and Sample Collection

A descriptive case control cross-sectional study was conducted in Bhaktapur district from July 2019 to January 2020. A total of 134 volunteers participated in the study, of which 67 were diabetic and 67 were nondiabetic. A total of 670 samples were collected, five each from different sites of each individual. The samples included oral wash, foot swab from interdigital space, midstream urine, hair shaft, and nail scrapings. Exactly 10 ml of sterile normal saline was given to individuals of both the study groups, and they were asked to rinse their oral cavity for 1 min, which was finally collected in sterile wide mouth container. Around 20–40 ml midstream urine sample was collected in a sterile wide-mouthed container. Cotton swab soaked in a sterile normal saline was used for collection of the sample from the interdigital space of toe. Hair shaft sample was collected by hair pluck method using forceps. Nail sample was collected by scraping the nail using a scalpel. All samples were collected aseptically and were transported to laboratory for processing within two hours of collection at the Department of Medical Laboratory Technology, JF Institute of Health Sciences, Hattiban, Lalitpur. Consent and history were taken before sample collection. Volunteer participants having recent history of antibiotic therapy, under immunosuppressive drugs, having cancer and harmful oral habits were excluded from study.

### 2.2. Processing of Samples

For isolation of fungi, samples were inoculated in Sabouraud dextrose agar (SDA) (HiMedia, Mumbai, India) containing chloramphenicol 50 mg/L concentration, incubated at 370°C and at 250°C for 7–14 days under aerobic conditions. The isolated colonies were further subcultured on Cornmeal Agar (HiMedia, Mumbai, India) containing 1% Tween 80 by applying Dalmau Plate Method for the cultivation of fungi and for the study of *Candida* species for chlamydospore production after incubation for 24–48 hours at 25–30°C. Fungal colonies morphology were recorded. Each isolates were stained by Gram stain for the detection of yeast cells. Colony growth which showed hyphae spreading on the media was identified by using lactophenol cotton blue mount. Among the isolates, *Candida* species were identified by subculture on CHROM agar (HiMedia, Mumbai, India) at 37°C for 48 hours.

### 2.3. Antibiotic Resistance Pattern of *Candida* Species

*Candida* species isolated were transferred to broth media and the turbidity of fungal growth was compared with 0.5 McFarland Standard. Samples were evenly streaked onto Mueller–Hinton agar supplemented with 2% glucose and 5 *μ*g/ml methylene blue. For internal control, *C. albicans* ATCC 90028 and *C. tropicalis* ATCC 750 were used. The zone of inhibition around the disc was measured after incubating the plates at 37°C for 24 hours in aerobic condition, and antibiotic susceptibility testing was performed as per the Clinical and Laboratory Standards Institute (CLSI) M44-A guidelines.

### 2.4. Data Analysis

Data was entered and analyzed by using SPSS software version 21. Independent *t*-test and linear regression analysis were applied and *p* value <0.05 was taken as significant.

## 3. Results

### 3.1. Status of Fungi Growth in Diabetic and Nondiabetic Study Groups

Female participants were higher in number as compared with male participants. In diabetic subjects, 38 (56.8%) were female and 29 (43.2%) were male, whereas, in nondiabetic subjects, 40 (59.8%) were female and 27 (40.3%) were male. Out of 670 samples, 130 (19.4%) samples showed fungal growth. The prevalence of fungi amongst diabetics was 114 (34.0%), and in nondiabetics, it was 16 (4.7%). In both diabetic and nondiabetic groups, the most frequently isolated fungus was *Candida* species 75 (57.6%), followed by *Aspergillus* species 37 (28.4%), *Trichophyton* species 14 (10.7%), *Mucor* species 3 (2.3%), and *Rhizopus* species 1 (0.8%). The number of fungi isolated was higher in diabetic population than the control group. Among diabetic individuals, the highest number of growths was observed in oral wash sample 45 (67.2%), followed by toe swab 29 (43.3%), midstream urine 22 (32.8%), hair samples 10 (14.9%), and nail samples 8 (11.9%). In nondiabetic individuals, oral wash and toe samples had a prevalence of 5 (7.5%) in each, while no growth was observed from midstream urine. Out of 75 *Candida* species isolates, 35 (46.6%) were *C. albicans*, followed by 17 (22.6%) *C. krusei*, 12 (16.0%) *C. glabrata*, and 11 (14.6%) *C. tropicalis* [11]. Similarly, out of 37 *Aspergillus* species, *A. nidulans* was found in 13 (35.1%), followed by *A. flavus* in 11 (29.7%), *A. niger* in 8 (21.6%), and *A. fumigatus* in 5 (13.5%). On comparing both groups for fungal growth, samples of oral wash, toe, and urine showed significantly higher prevalence among the diabetic population (*p* value < 0.05); difference is as shown in Table 1.

### 3.2. Relation between Age Group and Distribution of Fungal Isolates in Both Groups

In both study groups, higher numbers of pathogenic fungi were found in individuals in the age group between 46 and 55 years, followed by the age of more than 56 years and the age group between 36 and 46 years. No fungal isolates were observed in the age group of less than 35 years, except from hair samples. *P* value was significant (<0.05) in samples collected from the oral, toe, and urine, which is shown in Table 2.

### 3.3. Relation between Education Levels and Prevalence of Fungi

In this study, uneducated study subjects were higher in number (74), followed by lower secondary level [14], bachelor level [12], and primary level [10]. Uneducated subjects showed the highest fungal prevalence, which was 44.6%, followed by lower secondary level 15.3% and primary level 12.3%. Surprisingly, no fungi were isolated from the participants having a master degree and more qualifications. *P* value was found significant (<0.05) in the sample collection from oral, toe, and urine, as shown in Table 3.

### 3.4. Antibiotic Susceptibility Pattern of *Candida* Species

**Figure 1:**
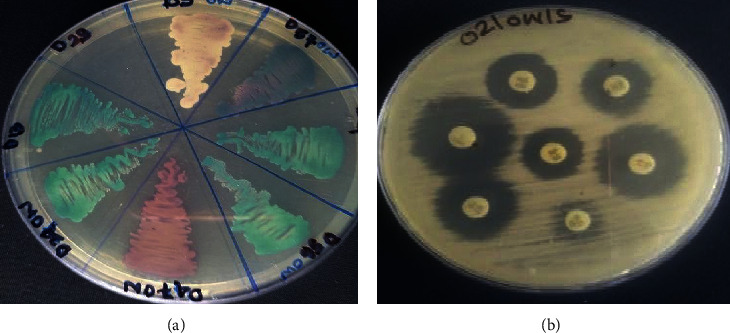
(a) Growth of *Candida* species in CHROMagar medium; (b) antibiotic susceptibility testing.

Of the total isolates, *Candida* species were isolated in 75 samples and were subjected to antibiotic sensitivity test (Figure 1(b)). Under standard in vitro conditions, 36.8% [15] isolates were resistant to fluconazole and 28.9% [16] were resistant to ketoconazole, while 10.5% [8] were resistant to miconazole. All isolates were sensitive to nystatin. Higher resistance was observed in *C. albicans*, followed by C *tropicalis*, *C. glabrata*, and *C. krusei* as shown in Table 4.

## 4. Discussion

Diabetes increases the risk of infection and damages multiple organs, which may affect the ability of protection against variety of pathogens. Poor glycemic control and chronic diabetes mellitus cause several complications like micro- and macrovascular complications, diabetic foot ulcers, eye infection, nephritis, and nerve infections, which are responsible for high morbidity and mortality [14, 17]. According to GAFFI, fungal infection kills more people than tuberculosis or malaria. Annually, approximately 11.5 million people are severely affected and more than 1.5 million die due to fungal infection. Fungal infections are not taken seriously in our community as a public health concern [1, 18]. Globally, 422 million are affected by diabetes and 1.6 million people lose their lives annually [2]. Hyperglycemic state causes immune dysfunction which leads to local and systemic infection due to overgrowth of microflora and causes an opportunistic infection. Uncontrolled diabetes allows fungal colonization in epithelial cells by sequentially increasing the number of receptors for colonization. Meanwhile, glucose, maltose, and sucrose boost the adhesion and suppression of the killing capacity of neutrophils [19]. Previous studies have shown that the incidence of fungal infection is higher in diabetic patients in comparison with nondiabetics. High blood sugar promotes the binding of fungus to the host cell surface, and high glucose in salivary secretions with low pH, poor oral hygiene, and very low salivary secretions also allow the growth of more than 50 *Candida* species colonies in the oral cavity [20].

In this study, the prevalence of fungus was observed to be 19.4%, which is similar to a recent study from India, in which the prevalence was 17.4% [13]. We found that the prevalence of fungal colonization in diabetics is eight times higher than that in nondiabetic participants, that is, 34.0% in diabetics and 4.7% in nondiabetics. Colonization in male and female was found to be in an equal proportion for both the study groups. Several studies have shown that the colonization rate of fungi in patients with diabetes is higher than in the nondiabetic individuals, that is, 84.0% and 27.0%, respectively [21]. Similarly, *Candida* species were isolated more frequently in diabetic population as compared with nondiabetic population. *C. albicans* among diabetics was 68.9% and 40.0% among healthy individuals [22]. However, another study found that there are no significant differences in *Candida* species isolated from diabetic individuals in comparison with healthy individuals [23].

In this study, *Aspergillus* species accounted for 28.4% prevalence, among which *A. nidulans* was 10%, followed by 8.46% of *A. flavus* and 3.84% *A. niger* of the total. A study from India also found *A. flavus* and *A. niger* in diabetic patients [11]. Prevalence of *Trichophyton* was 10.7%, which is similar to a study conducted in Portugal (14.3%) [16]. In the present study, we used CHROM agar for differentiation of isolated *Candida* species in standard in vitro conditions. CHROM agar had been used as selective and differential media for isolation of *Candida* species according to its colony color (Figure 1(a)). Various authors noted that CHROM agar has very good sensitivity and specificity for *C. albicans* ranging from 96.55% to 100% and from 96.42% to 100%, respectively [9, 24–26]. However, sensitivity and specificity are less when identifying *C. tropicalis*, where sensitivity ranged from 66.7% to 100%, and specificity, from 78.8% to 100%. *C. krusei* has sensitivity and specificity of 100% [24, 26]. Another study also showed that CHROM agar has a similar positivity as PCR-RFLP test method for *Candida* species differentiation.

In a study carried out on nondiabetic patients, it was found that there is no significant relationship between fungal infection in nails and level of education. It depended upon manual handwork practice, occupation and quality of life, socioeconomic status, gender, and age [27]. However, our study shows that there are no fungal isolates from participants with a master level of education in both study subjects.

Notwithstanding, the use of antibiotics for the treatment of bacterial infection increases the risk of fungal infection in hosts. For treatment of severe *Candida* infection, three classes of drugs (azoles, echinocandins, and amphotericin B) have been mainly used. According to the Center for Disease Control and Prevention, USA, nearly 7.0% of fungal bloodstream infection cases developed drug resistance [15]. In this study, out of 75 *Candida* species, 37.3% isolates were resistant to fluconazole, followed by 29.3% to ketoconazole and 10.6% to itraconazole. Other studies have also reported resistance of *Candida* spp. to flucytosine, ketoconazole, miconazole, and econazole, and the rate of resistance is higher in diabetic than nondiabetic individuals [22, 23]. In our study, fluconazole drug resistance in *Candida* species was 37.3%, which is higher than that in the study done in India and Singapore, that is, 9.3% and 3.2%, respectively [28]. Out of 75 isolates, 29.3% were resistant to ketoconazole, followed by 10.6% to miconazole and 8.0% to voriconazole. The cause for high resistance is unclear; perhaps, it could be due to the easy availability and increased use of drugs, irrational use, consumption without clinician's consultation, selling drugs without prescription, and limited diagnostic centers for fungus culture and identification in Nepal. In addition, it is also believed that fluconazole is one of the safest and effective drugs for topical and oral use in local and systemic fungal infections.

Fungal infection in diabetic patients causes serious morbidity and mortality. To minimize the burden of diseases, there is a need of proper use of prophylaxis and precise diagnosis of infectious disease, selection of proper drug and antibiotics, health hygiene awareness, and regular communication with patients. Most of the hospitals and laboratories in Nepal do not have the facility and required capacity to identify and test for antifungal drugs resistance. Developing countries have a lack of reliable, cost-effective diagnostic tools and limited therapeutic options. Increase in the morbidity of diabetes and the development of drug resistance among pathogenic fungi is a major threat to health and economy. The need for proper diagnosis of fungal diseases and availability of effective therapeutic agents at a low cost is a must for decreasing the morbidity. Moreover, diabetic patients need to be counseled about the need for proper hygiene, sanitation, and healthy living.

## 5. Conclusion

Diabetic patients are more prone to fungal colonization in comparison with nondiabetic individuals. The spread of fluconazole and ketoconazole-resistant *Candida* species is rising at an alarming rate in the community. Thus, there is a need to increase awareness regarding fungal infection and its impact on health in the community to reduce the burden of disease.

## Figures and Tables

**Table 1:** Prevalence of fungi in diabetic and nondiabetic individuals from different samples.

Fungi isolates	Oral wash		Toe sample		Urine sample		Nail sample		Hair sample	
	DM (*n* = 67)	NDM (*n* = 67)	DM (*n* = 67)	NDM (*n* = 67)	DM (*n* = 67)	NDM (*n* = 67)	DM (*n* = 67)	NDM (*n* = 67)	DM (*n* = 67)	NDM (*n* = 67)
*Candida albicans*	20 (44.4%)	2 (40.0%)	7 (24.1%)	1 (20.0%)	5 (22.7%)	0 (0.0%)	0 (0.0%)	0 (0.0%)	0 (0.0%)	0 (0.0%)
*Candida glabrata*	5 (11.1%)	0 (0.0%)	5 (17.2%)	1 (20.0%)	1 (4.5%)	0 (0.0%)	0 (0.0%)	0 (0.0%)	0 (0.0%)	0 (0.0%)
*Candida tropicalis*	4 (8.9%)	0 (0.0%)	2 (6.9%)	0 (0.0%)	5 (22.7%)	0 (0.0%)	0 (0.0%)	0 (0.0%)	0 (0.0%)	0 (0.0%)
*Candida krusei*	2 (4.4%)	1 (20.0%)	3 (10.3%)	0 (0.0%)	11 (50%)	0 (0.0%)	0 (0.0%)	0 (0.0%)	0 (0.0%)	0 (0.0%)
*Aspergillus flavus*	3 (6.7%)	2 (40.0%)	3 (10.3%)	2 (40.0%)	0 (0.0%)	0 (0.0%)	0 (0.0%)	0 (0.0%)	0 (0.0%)	1 (33.3%)
*Aspergillus fumigatus*	3 (6.7%)	0 (0.0%)	2 (6.9%)	0 (0.0%)	0 (0.0%)	0 (0.0%)	0 (0.0%)	0 (0.0%)	0 (0.0%)	0 (0.0%)
*Aspergillus niger*	2 (4.4%)	0 (0.0%)	2 (6.9%)	1 (20.0%)	0 (0.0%)	0 (0.0%)	0 (0.0%)	2 (66.7%)	0 (0.0%)	1 (33.3%)
*Aspergillus nidulans*	2 (4.4%)	0 (0.0%)	0 (0.0%)	0 (0.0%)	0 (0.0%)	0 (0.0%)	4 (50.0%)	0 (0.0%)	6 (60.0%)	1 (33.3%)
*Mucor* species	3 (6.7%)	0 (0.0%)	0 (0.0%)	0 (0.0%)	0 (0.0%)	0 (0.0%)	0 (0.0%)	0 (0.0%)	0 (0.0%)	0 (0.0%)
*Rhizopus* species	1 (2.2%)	0 (0.0%)	0 (0.0%)	0 (0.0%)	0 (0.0%)	0 (0.0%)	0 (0.0%)	0 (0.0%)	0 (0.0%)	0 (0.0%)
*Trichophyton* species	0 (0.0%)	0 (0.0%)	5 (17.2%)	0 (0.0%)	0 (0.0%)	0 (0.0%)	4 (50.0%)	1 (33.3%)	4 (40.0%)	0 (0.0%)
Total	45	5	29	5	22	0	8	3	10	3
*P* value	0.00		0.00		0.00		0.526		0.372	

**Table 2:** Relation between age group and distribution of fungal isolates in study subjects.

Sites	Age group (in year)	DM	NDM	*P* value
Oral samples	Less than 35	0 (0.0%)	0 (0.0%)	0.00
	36 to 45	6 (13.3%)	1 (20.0%)	
	46 to 55	21 (46.7%)	1 (20.0%)	
	More than 56	18 (40.0%)	3 (60.0%)	

Toe samples	Less than 35	0 (0.0%)	0 (0.0%)	0.00
	36 to 45	5 (17.2%)	1 (20.0%)	
	46 to 55	10 (34.5%)	2 (40.0%)	
	More than 56	14 (48.3%)	2 (40.0%)	

Urine samples	Less than 35	0 (0.0%)	0 (0.0%)	0.00
	36 to 45	2 (9.1%)	0 (0.0%)	
	46 to 55	12 (54.5%)	0 (0.0%)	
	More than 56	8 (36.4%)	0 (0.0%)	

Nail samples	Less than 35	0 (0.0%)	0 (0.0%)	0.469
	36 to 45	1 (12.5%)	1 (33.3%)	
	46 to 55	4 (50.0%)	0 (0.0%)	
	More than 56	3 (37.5%)	2 (66.7%)	

Hair samples	Less than 35	0 (0.0%)	1 (33.3%)	0.439
	36 to 45	2 (20.0%)	0 (0.0%)	
	46 to 55	4 (40.0%)	1 (33.3%)	
	More than 56	4 (40.0%)	1 (33.3%)	

**Table 3:** Relation between education levels and the prevalence of fungi in diabetic and nondiabetic groups.

	Oral sample		Toe sample		Urine sample		Nail sample		Hair sample	
	DM	NDM	DM	NDM	DM	NDM	DM	NDM	DM	NDM
Uneducated	21 (46.7%)	5 (100.0%)	13 (43.3%)	4 (80.0%)	6 (27.3%)	0 (0.0%)	3 (37.5%)	2 (66.7%)	3 (30.0%)	2 (66.7%)
Preprimary level	1 (2.2%)	0 (0.0%)	1 (3.3%)	0 (0.0%)	2 (9.1%)	0 (0.0%)	2 (25.0%)	0 (0.0%)	2 (20.0%)	0 (0.0%)
Primary level	6 (13.3%)	0 (0.0%)	4 (13.3%)	0 (0.0%)	3 (13.6%)	0 (0.0%)	0 (0.0%)	0 (0.0%)	2 (20.0%)	1 (33.3%)
Lower secondary level	9 (20.0%)	0 (0.0%)	5 (16.7%)	1 (20.0%)	3 (13.6%)	0 (0.0%)	1 (12.5%)	0 (0.0%)	1 (10.0%)	0 (0.0%)
Secondary level	3 (6.7%)	0 (0.0%)	1 (3.3%)	0 (0.0%)	2 (9.1%)	0 (0.0%)	0 (0.0%)	0 (0.0%)	0 (0.0%)	0 (0.0%)
Higher secondary level	3 (6.7%)	0 (0.0%)	4 (13.3%)	0 (0.0%)	4 (18.2%)	0 (0.0%)	1 (12.5%)	1 (33.3%)	1 (10.0%)	0 (0.0%)
Bachelor level	2 (4.4%)	0 (0.0%)	2 (6.7%)	0 (0.0%)	2 (9.1%)	0 (0.0%)	1 (12.5%)	0 (0.0%)	1 (10.0%)	0 (0.0%)
Master level	0 (0.0%)	0 (0.0%)	0 (0.0%)	0 (0.0%)	0 (0.0%)	0 (0.0%)	0 (0.0%)	0 (0.0%)	0 (0.0%)	0 (0.0%)
Total	45	5	30	5	22	0	8	3	10	3
*p* value	0.00		0.00		0.00		0.802		0.603	

**Table 4:** Antibiotic resistance pattern of *Candida* species isolated from both groups.

Antifungal agents	*Candida* species												Total *C.* species (*N* = 75)
	*C. albicans* (*N* = 35)			*C. krusei*(*N* = 17)			*C. tropicalis* (*N* = 11)			*C. glabrata* (*N* = 12)			
	S	I	R	S	I	R	S	I	R	S	I	R	R
Ketoconazole	14	2	19	13	3	1	10	1	0	8	2	2	22 (28.9%)
Voriconazole	33	1	1	13	4	0	7	2	2	8	1	3	6 (8.0%)
Amphotericin	7	28	0	7	10	0	3	7	1	5	7	0	1 (1.3%)
Itraconazole	24	8	3	14	2	1	11	0	0	10	1	1	5 (6.5%)
Miconazole	30	2	3	14	1	2	6	2	3	11	1	0	8 (10.5%)
Fluconazole	18	1	16	13	1	3	5	0	6	8	1	3	28 (36.8%)

*N* = number, *S* = sensitive, I = intermediate, and *R* = resistant.

## Data Availability

The data used to support the findings of this study are available from the corresponding author upon request. The authors agree that anyone interested can have access to the raw data from the research upon request to the authors. The data will be provided to anyone based on two conditions: upon use of the data, the authors should be acknowledged and also the paper needs to be cited. An agreement in the aforementioned situation will be reached.

## Abbreviations

C: *Candida*
GAFFI: Global Action Fund for Fungal Infections.
